# The Application of Preoperative Three-Dimensional Reconstruction Visualization Digital Technology in the Surgical Treatment of Hepatic Echinococcosis in Tibet

**DOI:** 10.3389/fsurg.2021.715005

**Published:** 2021-08-19

**Authors:** Jun Zhang, DAWA, Duojie Suolang, Yanming Lei, Jiayun Wang, Dunzhu Basang

**Affiliations:** ^1^Department of General Surgery, Peking University First Hospital, Beijing, China; ^2^Department of General Surgery, People's Hospital of Tibet Autonomous Region, Lhasa, China; ^3^Department of Imaging, People's Hospital of Tibet Autonomous Region, Lhasa, China; ^4^Department of Clinical Medicine, Capital Medical University, Beijing, China

**Keywords:** hepatic echinococcosis, preoperative three-dimensional reconstruction, surgical plan, 3D visualization reconstruction, Tibet

## Abstract

**Objective:** The present study aims to explore the application value of three-dimensional (3D) reconstruction technology in the preoperative evaluation of patients with complicated hepatic echinococcosis in Tibet.

**Methods:** A total of 200 patients with complicated hepatic echinococcosis, admitted to our hospital between May 2019 and December 2020, who underwent radical hepatectomy, were enrolled in the present study. The patients were randomly divided into a preoperative computer tomography group and a preoperative 3D reconstruction group. According to the imaging results, a surgical plan was formulated. A comparison was made between the two groups of the coincidence rate of the surgical plan and intraoperative and postoperative complications.

**Results:** The patients with hepatic echinococcosis who underwent 3D visualization reconstruction before surgery had a high compliance rate with the surgical plans and the operating time, the number of cases with blood flow blockage, the blood flow blockage time, intraoperative hemorrhage, and postoperative biliary fistulas were significantly lower.

**Conclusion:** The application of preoperative 3D visualization reconstruction in patients with complicated hepatic echinococcosis in Tibet could effectively improve surgical safety.

## Introduction

Echinococcosis is a common parasitic disease in Tibet. The liver is the organ with the highest incidence of echinococcosis (1), and radical hepatectomy is currently the most common treatment for hepatic echinococcosis. Patients with hepatic echinococcosis are usually late in making a hospital visit in Tibet. By the time it is discovered, the hydatid has often invaded the blood vessels and biliary system, and the intraoperative conditions are complicated. Moreover, Tibet is located in a plateau xarea, and patients have poor coagulation function and can easily hemorrhage during surgery, making it highly risky. Therefore, it is imperative to make a sound surgical plan before hepatectomy to increase the radical cure rate of hepatic echinococcosis and reduce postoperative complications and mortality.

In the past 10 years, new concepts such as “precision liver resection” and “planned liver resection” have continually been innovated and developed (2). Digital medical technology plays a vital role in improving predictability and controllability during the practice of hepatic surgery. It has become feasible to apply preoperative three-dimensional (3D) reconstruction visualization technology to the field of liver surgery, with the adoption of a digital surgical technology platform, to quantitatively analyze the liver anatomy and morphological characteristics of the lesion before an operation, carry out virtual liver resection, and implement a safe surgical plan to guide the surgery.

In the present study, preoperative 3D reconstruction technology was used for the first time in Tibet to design preoperative virtual surgery for patients with complicated hepatic echinococcosis. The study evaluates the application value of this technology in preoperative evaluation for hepatic echinococcosis in Tibet.

## Materials and Methods

### Study Subjects

A total of 200 patients with complicated hepatic echinococcosis admitted to our hospital between May 2019 and December 2020 to undergo radical hepatectomy were enrolled in the present study.

The inclusion criteria were patients with alveolar echinococcosis; patients with complicated cystic echinococcosis (cystic hydatid with an invasion of the vascular structures such as the main hepatic vein, the inferior vena cava behind the liver, and the Glisson sheath at the first hepatic hilar); patients with a history of surgery for hepatic echinococcosis; patients with a Child–Pugh score of grades A–B; patients who were willing and able to participate in all follow-ups.

The study was conducted in accordance with the Declaration of Helsinki (revised in 2013). The Ethics Committee of Peking University First Hospital approved this study (No.ME-TBHP-2013). Written consent was obtained from each patient.

### Methods

According to random numbers generated by a computer, the patients were randomly divided into a preoperative computer tomography (CT) group and a preoperative 3D reconstruction group. Each group was composed of 100 patients. Those with odd numbers were assigned to the preoperative CT group and those with even numbers to the preoperative 3D reconstruction group.

All patients had an enhanced abdominal CT scan before surgery. The information provided by the CT concerning the anatomy of the liver, and its adjacent relationship with the hydatid, was used to select the surgical plan for patients in the preoperative CT group. The preoperative 3D reconstruction was completed based on the CTs of the patients in the preoperative 3D reconstruction group. The flow in the liver ducts was observed by performing a 360-degree multi-axial rotation of the reconstructed model, zooming in and reducing the image, and transparentizing the reconstructed organ model. The size and shape of the hydatid and the neighboring relationship between the hydatid and important blood vessels in the liver were observed from multiple angles to understand whether there was any vascular invasion. At the same time, for patients in the preoperative 3D reconstruction group, preoperative virtual liver resection and calculation of the percentage of residual hepatic volume were performed to determine the surgical risk and the possibility of resection of the hydatid, as well as to make a surgical plan.

The gender, age, height, and weight of each patient were recorded. The preoperatively planned operation method, the actual intraoperative operation method, operation time, cases and times of hepatic blood flow blockage, cases with intraoperative blood transfusion and volume, the volume of intraoperative hemorrhage, and any postoperative complications including hemorrhage, biliary fistula, hepatic failure, incision and pulmonary infections, portal and deep vein thrombosis, abdominal and pleural effusions, or even death were recorded, along with the follow-up rate.

### Statistical Methods

SPSS 25.0 software was used for the data analysis. A *t*-test was used for prospective cohort analysis, and an exact probability test was carried out for a comparison of rates.

## Results

### General Characteristics

A total of 200 patients with complicated hepatic echinococcosis, who underwent radical hepatectomy, were involved in the study. They were comprised of 97 males and 103 females, aged 15–72 years, with an average age of 46.24 ± 11.45 years. There was no statistically significant difference in age, gender, height, or weight between the preoperative CT and preoperative 3D reconstruction groups. There was no statistical difference between the two groups in the proportion of patients with cystic and alveolar echinococcosis. There was no statistical difference between the two groups in the proportion of invasion with the portal vein, hepatic vein, or inferior vena cava by the hydatid (as shown in Table 1). All patients underwent surgical resection.

**Table 1:** The general characteristics of the patients between the preoperative CT group and the preoperative three-dimensional reconstruction group before surgery.

**Indicators**	**Results**		***P*-value**
	**Preoperative CT group**	**Preoperative 3D reconstruction group**	
Age (years)	40.1	40.5	>0.05
Gender (Male)	49	48	>0.05
Height (cm)	164.8	165.1	>0.05
Weight (kg)	61.2	63.4	>0.05
Cystic echinococcosis(Case)	66	63	>0.05
Invasion of the grade II and grade III branch of the portal vein(Case)	16	20	>0.05
Invasion of the hepatic vein(Case,%)	21	22	>0.05
Invasion of the Vena cava(Case, %)	1	3	>0.05
Previous surgery for echinococcosis(Case)	16	20	>0.05

### Intraoperative Condition

The patients in the preoperative 3D reconstruction group had a significantly higher rate of compliance with the surgical plan than the preoperative CT group did, and the difference was statistically significant (as demonstrated in Table 2). There was no statistical difference in the two surgical methods between the two groups (as shown in Table 3). The operation time, the volume of intraoperative hemorrhage, the number of cases with blood flow blockage, and blood blockage time in the preoperative 3D reconstruction group were significantly less than the preoperative CT group, and the differences were statistically significant. Compared with the preoperative CT group, the number of cases with blood transfusion and the volume of blood transfusion in the preoperative 3D reconstruction group was not statistically different, but these indicators were lower in the latter group (as illustrated in Table 4).

**Table 2:** The compliance rate of the surgical plan between the preoperative CT group and the preoperative three-dimensional reconstruction group.

**Groups**	**Number of cases was the same as the preoperative plan**	**Number of cases was different from the preoperative plan**	***P*-value**
Preoperative CT group	53	47	<0.05
Preoperative 3D reconstruction group	86	14	

**Table 3:** Comparison of the surgical mode between the preoperative CT group and the preoperative three-dimensional reconstruction group.

**Surgical mode**	**Results**		***P*-value**
	**Preoperative CT group**	**Preoperative 3D reconstruction group**	
Left hepatectomy	6	8	>0.05
Right hepatectomy	26	28	
Left trilobectomy	0	0	
Right trilobectomy	0	0	
Caudate lobectomy	0	0	
Left outer lobe resection	7	10	
Right outer lobe resection	0	0	
Right posterior lobe resection	3	0	
Middle lobectomy	0	0	
Partial liver resection	37	35	
Internal capsule removal	10	10	
Complete exocystectomy	0	0	
Subtotal external capsule resection	11	9	
Exploratory laparotomy	0	0	

**Table 4:** The intra-operative condition between the preoperative CT group and the preoperative three-dimensional reconstruction group.

**Indicators**	**Results**		***P*-value**
	**Preoperative CT group**	**Preoperative 3D reconstruction group**	
Operation time(min)	210	135	<0.05
Blockage of the hepatic blood flow(case)	83	50	<0.05
The average duration of the blood flow blockage into the liver(min)	30.1	18.2	<0.05
Intra-operative blood transfusion(Case,%)	16	10	>0.05
The volume of intra-operative blood transfusion (ml)	550	310	>0.05
Intra-operative hemorrhage(ml)	613	312	<0.05

### Postoperative Condition

The incidence of postoperative biliary fistula in the preoperative 3D reconstruction group was significantly lower than in the preoperative CT group, and the difference was statistically significant. The difference in the incidence of other complications was not statistically significant, but the incidence of pleural effusion in the preoperative 3D reconstruction group was lower than in the preoperative CT group (as shown in Table 5).

**Table 5:** Comparison of the postoperative complications between the preoperative CT group and the preoperative three-dimensional reconstruction group.

**Indicators**	**Results**		***P*-value**
	**Preoperative CT group**	**Preoperative 3D reconstruction group**	
**Complication**			
Main post-operative complications			
Hemorrhage requiring re-operation	0	0	>0.05
Biliary fistula requiring puncture and drainage	0	0	>0.05
Pleural effusion requiring puncture and drainage	2	0	>0.05
Abdominal effusion requiring puncture and drainage	0	0	>0.05
Post-operative hepatic failure	0	0	>0.05
Death	0	0	>0.05
Other complications			>0.05
Incision infection	0	3	>0.05
Pulmonary infection	6	6	>0.05
Deep vein thrombosis	0	0	>0.05
Portal vein thrombosis	0	0	>0.05
Biliary fistula requiring no treatment	16	3	<0.05
Abdominal effusion requiring no treatment	3	6	>0.05
Pleural effusion requiring no treatment	9	3	>0.05

### Case Example

A 22-year-old female patient was admitted with the complaint, “discovery of liver occupancy by B-ultrasonography for half a month,” and her Child–Pugh score was grade A.

**Figure 1:**
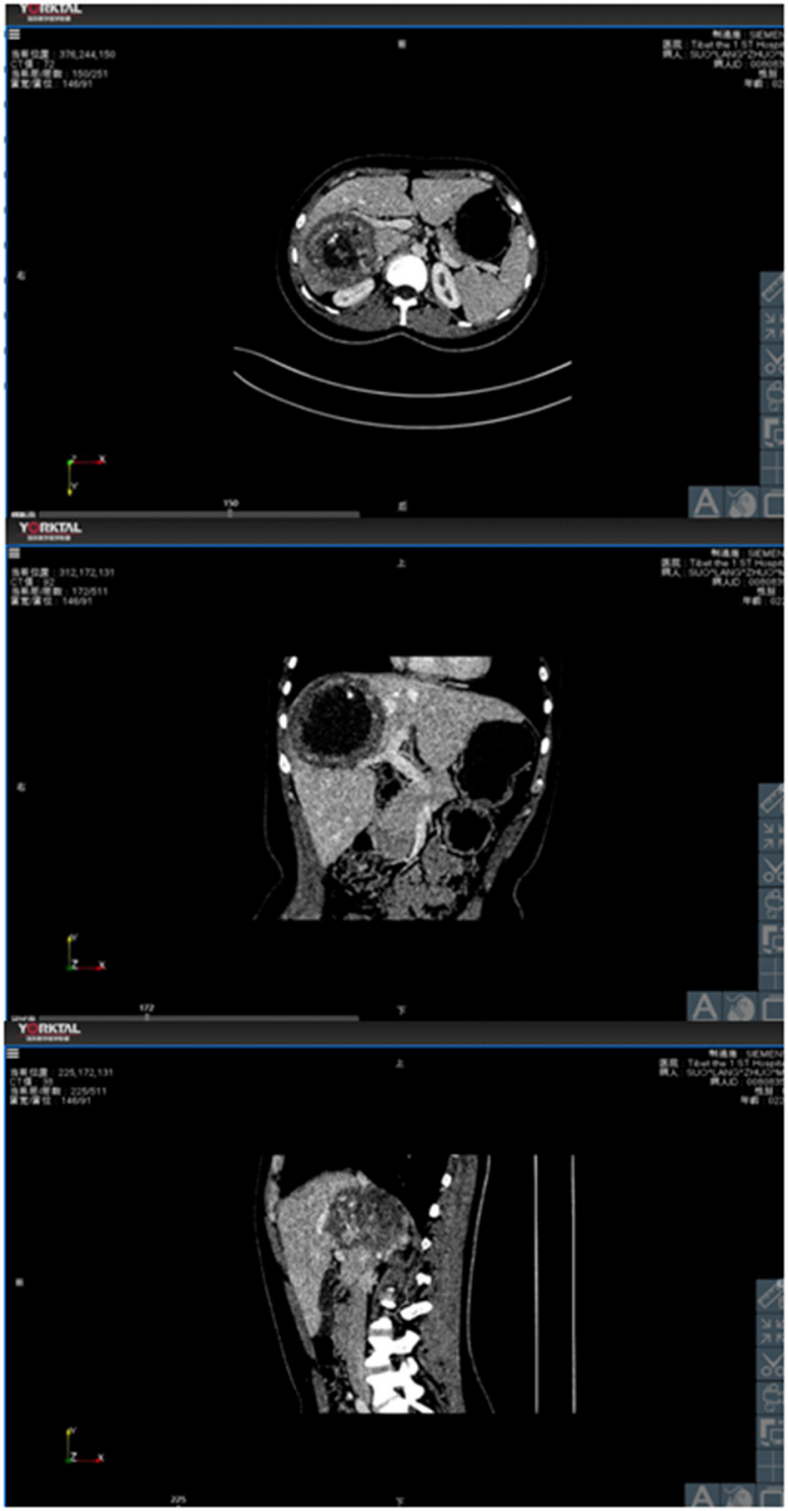
The relationship between hydatid and portal vein, hepatic vein and inferior vena cava demonstrated by the cross-section, coronal and sagittal planes in CTs.

**Figure 2:**
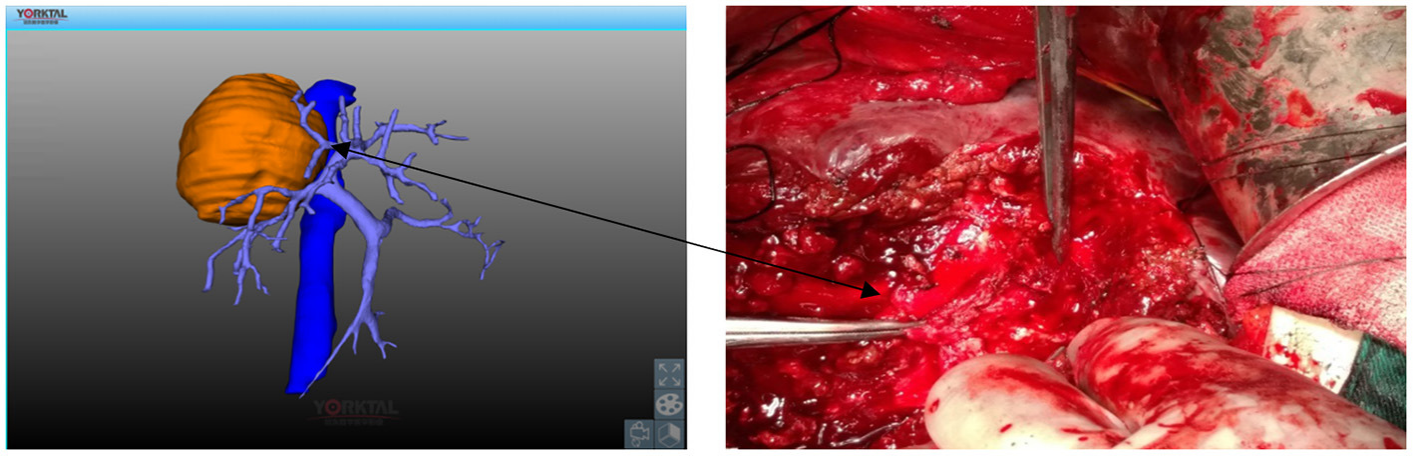
Invasion of the V segmental branch of the portal vein by hydatid. Solid line arrow: V segmental branch of the portal vein.

**Figure 3:**
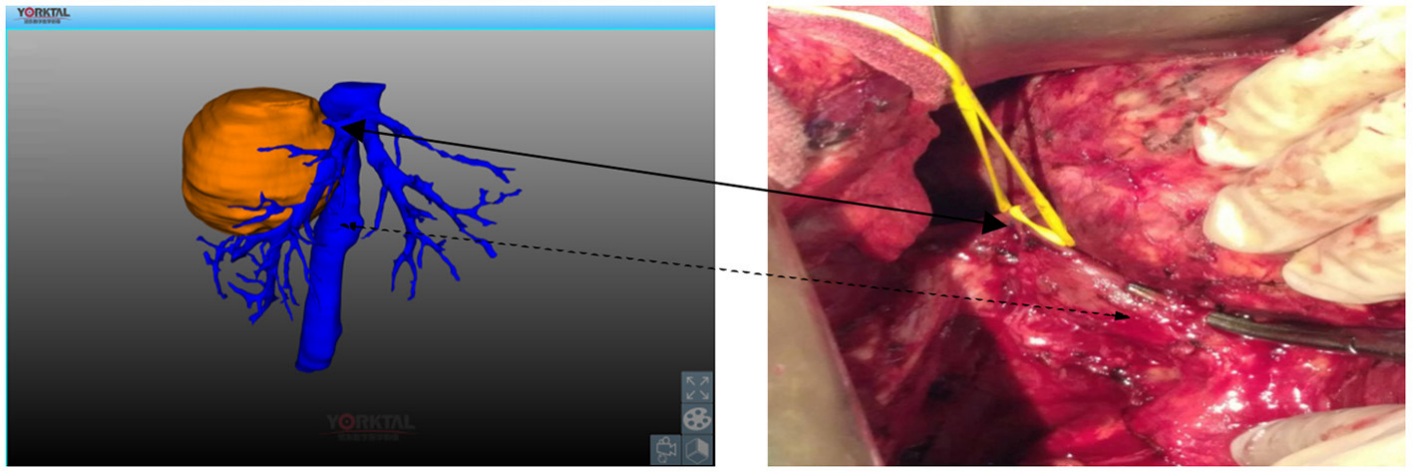
Invasion of the right hepatic vein and inferior vena cava by hydatid. Solid line arrow: right hepatic vein; Dashed arrow: inferior vena cava.

The preoperative enhanced CT revealed a hydatid in the right hepatic lobe (as shown in Figure 1), and the preoperative 3D reconstruction showed that the hydatid was invading segment V of the portal vein, right hepatic vein, and inferior vena cava. The preoperatively planned operation mode was right hepatectomy, partial resection, and repair of the inferior vena cava, and the operation methods were consistent with the preoperative plan. The intraoperative conditions were consistent with the conditions manifested by the preoperative 3D reconstruction. The segment V of the portal vein was visible during the operation (as illustrated in Figure 2). After the operation, the middle hepatic vein was exposed, and the right hepatectomy and repaired inferior vena cava were visible (as shown in Figure 3).

## Discussion

Hepatectomy is currently the most common method used for the treatment of hepatic echinococcosis (3, 4). There are no residual lesions after radical hepatectomy, and the prognosis is good. In combination with anti-hydatid drug treatment after surgery, a clinical cure can be achieved.

There are no obvious symptoms in the early stage of hepatic echinococcosis. The hydatid has often already invaded important blood vessels or the biliary system by the time patients are treated in Tibet. In addition, Tibet is located on a plateau, so due to chronic hypoxia, the function of blood coagulation in patients has been activated for a long time. Since the platelet and coagulation factor reserves are fewer, it is more likely that a patient from this type of area will develop a hemorrhage compared with a patient from the plains areas (5–7). In addition, radical hepatectomy for complicated hepatic echinococcosis often requires hilar blockage, removing a large amount of hepatic parenchyma, and even vascular reconstruction. However, massive removal of hepatic parenchyma may lead to postoperative hepatic failure and even cause postoperative death. Ferrero et al. pointed out that the residual hepatic volume was an independent risk factor for hepatic insufficiency after hepatectomy (8). Therefore, the key to avoiding serious postoperative complications is to carry out a comprehensive preoperative evaluation to determine the optimal surgical section for a precise liver resection (9–11). In Tibet, routine preoperative biochemical examinations, Child–Pugh scores, and indocyanine green 15-min tests are the primary methods used for the preoperative evaluation of liver function. However, abnormal serum bilirubin and hemodynamic stability will affect the accuracy of the above tests (12). The assessment of the residual hepatic volume is currently a common and reliable method before liver tumor resection. For normal livers, the ratio of residual hepatic volume to total hepatic volume is >0.3. For cirrhosis with hepatitis and abnormal liver function after liver chemotherapy, a ratio of residual hepatic volume to a total hepatic volume >0.5 can effectively prevent the occurrence of postoperative hepatic failure (13, 14). Previously, the evaluation of the total and residual hepatic volume was roughly estimated using the preoperative CT and other images, and the accuracy rate was relatively poor (15, 16). Preoperative 3D reconstruction visualization technology can automatically and accurately calculate the reconstructed total hepatic volume and volume of the hydatid, together with the estimated hepatic volume, residual hepatic volume, and the ratio of residual hepatic volume through simulated surgery. This can significantly reduce the probability of the occurrence of hepatic failure after radical hepatectomy (17).

Currently, the preoperative evaluation of hepatic echinococcosis in Tibet is based on preoperative images, such as CT and B-ultrasonography. Surgeons need to combine their own surgical experience with imaging knowledge to perform image synthesis and spatial imaging and establish and analyze the preoperative 3D reconstruction images in the brain to investigate the distribution of intrahepatic vasculature and the relationship with the hydatid. However, due to surgeons' varying degrees of surgical experience, their ability to read the pictures is also quite diverse, leading to different preoperative 3D reconstruction images being formed by several doctors concerning the same patient. Moreover, due to the inability of the human brain to maintain a long-term memory of the preoperative 3D reconstruction spatial imaginary structure, the preoperative evaluation is often inaccurate. What is more, even the evaluation results of several doctors can be quite different, resulting in accidents that force the operation to be stopped. However, preoperative 3D reconstruction visualization technology can accurately reconstruct the anatomical course and characteristics of each vascular system of the liver, as well as the relationship between the hydatid and the vascular system in the liver. More importantly, compared with the preoperative 3D reconstruction software that comes with CT, the preoperative 3D reconstruction visualization technology can delineate the estimated resection line and the scheduled resection range according to the hydatid position before the surgeon performs a virtual hepatic resection. During virtual cutting, any complications that may occur during the actual operation can be addressed, and modifications made accordingly. Finally, since the virtual procedure can be performed repeatedly, with the simulation and comparison of different surgical plans, the possibility of severe hemorrhage and other complications during the operation can be reduced, and a safe, reasonable, and comprehensive individualized treatment plan for the patient can be formulated.

## Conclusion

The present study shows that, for patients with hepatic echinococcosis, preoperative 3D reconstruction can be used to individually and accurately formulate a preoperative plan with a high compliance rate, a reduced operation time, fewer cases with blood flow blockage, reduced blood flow blockage time, and a reduced volume of intraoperative hemorrhage, together with a lower incidence of postoperative biliary fistula and a significant improvement in the safety of surgery. Therefore, this technology can contribute to the improved diagnosis and treatment of complicated hepatic echinococcosis in Tibet.

## Data Availability Statement

The original contributions presented in the study are included in the article/supplementary material, further inquiries can be directed to the corresponding author/s.

## Author Contributions

JZ, DS, and JW: conception and design of the research. JZ, JW, and DB: writing of the manuscript. D, YL, and DB: acquisition of data. D, DS, and YL: analysis and interpretation of the data and statistical analysis. JZ: obtaining financing and critical revision of the manuscript for intellectual content. All authors contributed to the article and approved the submitted version.

## Conflict of Interest

The authors declare that the research was conducted in the absence of any commercial or financial relationships that could be construed as a potential conflict of interest.

## Publisher's Note

All claims expressed in this article are solely those of the authors and do not necessarily represent those of their affiliated organizations, or those of the publisher, the editors and the reviewers. Any product that may be evaluated in this article, or claim that may be made by its manufacturer, is not guaranteed or endorsed by the publisher.
