# Corrigendum: The Application of Preoperative Three-Dimensional Reconstruction Visualization Digital Technology in the Surgical Treatment of Hepatic Echinococcosis in Tibet

**DOI:** 10.3389/fsurg.2021.772488

**Published:** 2021-10-19

**Authors:** Jun Zhang, DAWA, Duojie Suolang, Yanming Lei, Jiayun Wang, Dunzhu Basang

**Affiliations:** ^1^Department of General Surgery, Peking University First Hospital, Beijing, China; ^2^Department of General Surgery, People's Hospital of Tibet Autonomous Region, Lhasa, China; ^3^Department of Imaging, People's Hospital of Tibet Autonomous Region, Lhasa, China; ^4^Department of Clinical Medicine, Capital Medical University, Beijing, China

**Keywords:** hepatic echinococcosis, preoperative three-dimensional reconstruction, surgical plan, 3D visualization reconstruction, Tibet

The second author's name was incorrectly spelled as “Jinmei Dawa.” The correct spelling is “DAWA.”

As a result, the **Author Contributions** section has been updated to change the initials “JD” to “D”. The updated statement is below.

“JZ, DS, and JW: conception and design of the research. JZ, JW, and DB: writing of the manuscript. D, YL, and DB: acquisition of data. D, DS, and YL: analysis and interpretation of the data and statistical analysis. JZ: obtaining financing and critical revision of the manuscript for intellectual content. All authors contributed to the article and approved the submitted version.”

The authors apologize for this error and state that this does not change the scientific conclusions of the article in any way. The original article has been updated.

## Publisher's Note

All claims expressed in this article are solely those of the authors and do not necessarily represent those of their affiliated organizations, or those of the publisher, the editors and the reviewers. Any product that may be evaluated in this article, or claim that may be made by its manufacturer, is not guaranteed or endorsed by the publisher.

